# A mutation in the dynein heavy chain gene compensates for energy deficit of mutant SOD1 mice and increases potentially neuroprotective IGF-1

**DOI:** 10.1186/1750-1326-6-26

**Published:** 2011-04-26

**Authors:** Anissa Fergani, Judith Eschbach, Hugues Oudart, Yves Larmet, Birgit Schwalenstocker, Albert C Ludolph, Jean-Philippe Loeffler, Luc Dupuis

**Affiliations:** 1 Inserm U692, Laboratoire de Signalisations Moléculaires et Neurodégénérescence, Strasbourg, F-67085 France; 2 Université de Strasbourg, Faculté de Médecine, UMRS692, Strasbourg, F-67085 France; 3 DEPE, IPHC, Strasbourg, France; 4 Department of Neurology, University of Ulm, Ulm, Germany

## Abstract

**Background:**

Amyotrophic lateral sclerosis (ALS) is a fatal neurodegenerative disease characterized by a progressive loss of motor neurons. ALS patients, as well as animal models such as mice overexpressing mutant SOD1s, are characterized by increased energy expenditure. In mice, this hypermetabolism leads to energy deficit and precipitates motor neuron degeneration. Recent studies have shown that mutations in the gene encoding the dynein heavy chain protein are able to extend lifespan of mutant SOD1 mice. It remains unknown whether the protection offered by these dynein mutations relies on a compensation of energy metabolism defects.

**Results:**

SOD1(G93A) mice were crossbred with mice harboring the dynein mutant *Cramping *allele (*Cra*/+ mice). Dynein mutation increased adipose stores in compound transgenic mice through increasing carbohydrate oxidation and sparing lipids. Metabolic changes that occurred in double transgenic mice were accompanied by the normalization of the expression of key mRNAs in the white adipose tissue and liver. Furthermore, Dynein *Cra *mutation rescued decreased post-prandial plasma triglycerides and decreased non esterified fatty acids upon fasting. In SOD1(G93A) mice, the dynein *Cra *mutation led to increased expression of IGF-1 in the liver, increased systemic IGF-1 and, most importantly, to increased spinal IGF-1 levels that are potentially neuroprotective.

**Conclusions:**

These findings suggest that the protection against SOD1(G93A) offered by the *Cramping *mutation in the dynein gene is, at least partially, mediated by a reversal in energy deficit and increased IGF-1 availability to motor neurons.

## Background

Amyotrophic lateral sclerosis (ALS) is a neurodegenerative disease characterized by a progressive loss of motor neurons in the motor cortex, brainstem and spinal cord. ALS patients develop progressive muscle weakness and paralysis leading to death 3 to 5 years after first symptoms. Despite most cases of ALS occur sporadically, 5% are genetically inherited. Out of these familial forms of ALS, a subset is caused by mutations in the gene encoding the Cu/Zn-superoxide dismutase 1 (SOD1).

Studies on patients and transgenic mutant SOD1 mice showed that ALS-linked neurodegeneration was usually associated with defects in energy homeostasis [1]. Indeed, mutant SOD1 mice show a pronounced hypermetabolism characterized by weight loss, increased oxygen consumption and an increased use of lipids stores [2,3]. In sporadic ALS patients, hyperlipemia was associated with increased survival [4,5]. The mechanisms by which such changes in energy metabolism participate to motor neuron degeneration remain unknown.

Recent studies have shown that mutations in cytoplasmic dynein heavy chain gene protect motor neurons against death and extend survival of mutant SOD1 mice [6-8]. Cytoplasmic dynein is the major molecular motor responsible for retrograde axonal transport in neurons. Three different mutations in the dynein heavy chain gene, respectively called "*legs at odd angles*" (*Loa*), "*Cramping*" (*Cra*) and "*Sprawling*" (*Swl*) have been identified in ENU-induced mouse strains [9,10]. All three mutations lie in the stem domain of dynein heavy chain and the *Loa *mutation disrupts, at least partially, the dynein complex [11]. In the nervous system, these mutations lead to perinatal proprioceptive neuropathy [8,10,12]. *Cra*/+ and *Loa*/+ mice are hyperactive [13,14], and this is associated, at least in *Cra*/+ mice with striatal atrophy and compromised neurite outgrowth of striatal neurons [13]. Besides the nervous system, *Cra*/+ and *Loa*/+ mice display a major phenotype in adipose tissues [15]. Indeed, *Cra*/+ and *Loa*/+ mice show strikingly increased adipose stores, along with compromised thermogenesis. This is most likely due to defective stimulated lipolysis [15].

The extension in lifespan offered by mutations in dynein heavy chain gene has been attributed to several mechanisms, including compensation of axonal transport defects [6] and mitochondrial dysfunction [16], or decreased excitotoxic glutamate input to motor neurons due to degeneration of proprioceptive neurons [8]. In this report we provide evidence that the dynein mutation is able to revert the energy deficit characteristic of ALS in mutant SOD1 mice. Interestingly, this is associated with increased hepatic IGF-1 expression and increased spinal IGF-1. Taking into account these results, we propose that the mutation in dynein is able to provide neuroprotection against SOD1-ALS through complementary pathways.

## Results

### Dynein mutation compensates for energy deficit of early symptomatic transgenic SOD1(G93A) mice

**Figure 1:**
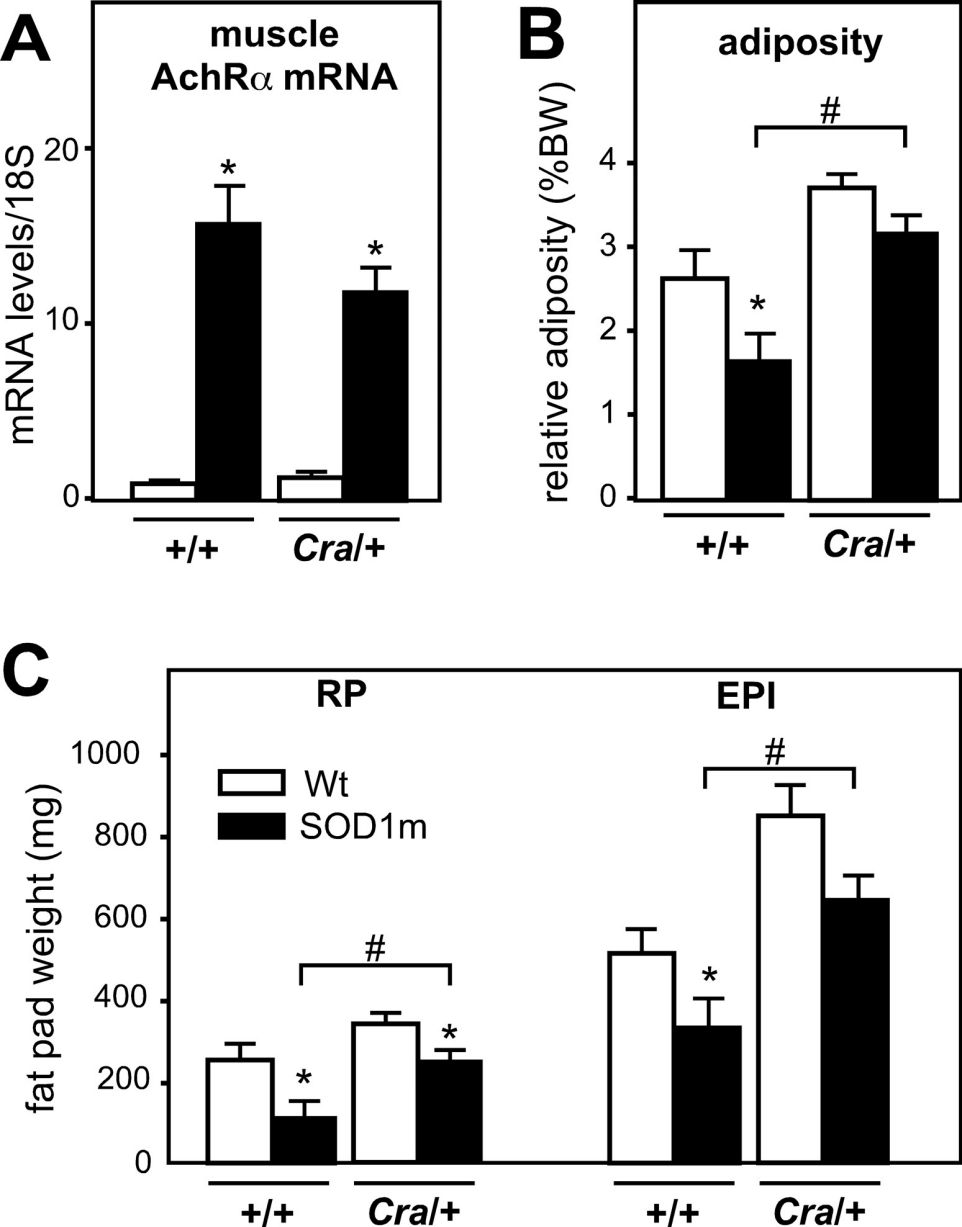
**Dynein mutation increased adipose stores in early symptomatic SOD1(G93A) mice**. A- mRNA levels of alpha subunit of the nicotinic acetylcholine receptor (AchRα) in gastrocnemius muscles of wild type (+/+) and dynein mutant mice (*Cra*/+) bearing SOD1(G93A) transgene (SOD1m, black columns) or not (Wt, empty columns) *P < 0.05 *versus *Wt. mRNA levels were standardized using 18S ribosomal RNA as a control. N = 9 mice per group. B-C- Relative weight of epididimary (EPI) and retroperitoneal (RP) white adipose tissue fat pad with regard to body weight (B). The panel C shows absolute weight (in mg) of EPI and RP in the same mice than in A. *P < 0.05 *versus *Wt; #, p < 0.05 as indicated. N = 9 mice per group.

We hypothesized that the protection offered by dynein mutation against SOD1(G93A) neurodegeneration was linked to a compensation of the energy deficit of SOD1(G93A) mice. To test this hypothesis, we crossbred SOD1(G93A) mice with *Cra*/+ mice and studied the energetic physiology of compound heterozygotes before any obvious motor symptoms. At 16 weeks of age, SOD1(G93A) nor *Cra*/SOD1(G93A) mice did not show obvious clinical signs such as gait impairment but both groups of SOD1(G93A) mice were lighter than wild type littermates. Body weight deficit of *Cra*/SOD1(G93A) mice was less than SOD1(G93A) mice (not shown) as previously shown [7] and both groups displayed similar upregulation of the denervation marker AchRα in the gastrocnemius muscle compared to non-SOD1(G93A) animals (Figure 1A). This early symptomatic age was selected for further studies. Both groups of SOD1(G93A) mice showed decreased weights of epididymary and retroperitoneal fat pads, but *Cra*/SOD1(G93A) fat pads were larger than that of SOD1(G93A) mice (Figure 1B-C). This was not due to increased food intake since all groups displayed similar food intake (data not shown). In all, these results demonstrate that the *Cramping *mutation in the dynein heavy chain gene compensated partially for SOD1(G93A) energy deficit in early symptomatic animals.

### Dynein mutation does not modify SOD1(G93A) hypermetabolism

**Figure 2:**
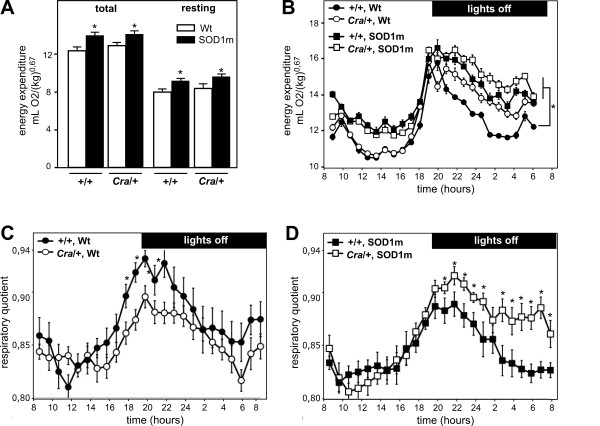
**Dynein mutation does not decrease global hypermetabolism but shifts energy metabolism of SOD1(G93A) mice towards carbohydrate use**. A- Total (left panel) and resting (right panel) energy expenditure of wild type (+/+) and dynein mutant mice (*Cra*/+) bearing SOD1(G93A) transgene (SOD1m, black columns) or not (Wt, empty columns) *P < 0.05 *versus *Wt. Note that SOD1(G93A) mice are hypermetabolic and that the dynein mutant genotype has no effect on this hypermetabolism. N = 9 mice per group. B- Energy expenditure of the same mice than in A as a function of time. Mice bearing a *Cra *dynein mutation are denoted by empty symbols, and their corresponding controls by a filled symbol. Mice bearing a SOD1(G93A) (SOD1m) transgene are labeled by a squared symbol and their controls by a circle. Note that in the diurnal period, there are no differences between +/+ and *Cra*/+ mice but that the presence of a SOD1(G93A) transgene increases energy expenditure. On the contrary, during nocturnal activity period, the presence of either *Cra*/+ mutation or SOD1(G93A) transgene increases energy expenditure. N = 9 mice per group. C-D- Respiratory quotient of the same mice than in A. The four groups of mice are shown in two graphs for clarity reasons. Mice non transgenic for SOD1(G93A) (SOD1m) are shown in panel C (filled symbols, +/+; empty symbols, *Cra*/+). Mice transgenic for SOD1(G93A) are shown in panel D (filled symbols, +/+; empty symbols, *Cra*/+). Note that the presence of a dynein mutation increases respiratory quotient in otherwise wild type mice, and even more potently in SOD1(G93A) mice.

We next determined whether the *Cramping *dynein mutation compensated for energy deficit through decreased SOD1(G93A) linked hypermetabolism. For this, we measured energy expenditure of the four groups of mice. As previously shown [3], SOD1(G93A) animals had a 15-20% increase in resting energy expenditure (Figure 2A), and this hypermetabolism was unchanged by *Cramping *dynein mutation. Furthermore, SOD1(G93A) associated increased total energy expenditure was also unchanged (Figure 2A) suggesting that the compensation in energy deficit was not provided by, for instance, decreased activity. Thus, the protective potential of *Cramping *dynein mutation was independent of a direct modulation of global hypermetabolism. Closer examination of indirect calorimetry results revealed however that oxygen consumption of SOD1(G93A) and *Cra*/SOD1(G93A) but also *Cra*/+ mice were increased during the nocturnal period as compared with +/+ littermates (Figure 2B). The respiratory quotient of *Cra*/+ and *Cra*/SOD1(G93A) mice was higher than that of +/+ and SOD1(G93A) mice at the same period (Figure 2C-D). Since oxidation of carbohydrates leads to a respiratory quotient of 1, while oxidation of lipids leads to a respiratory quotient of 0.7 [17], these results show that the dynein mutation leads to a shift in nutrient use during activity towards preferential carbohydrate oxidation, and thus sparing of lipids and increased fat pad weights.

### Dynein mutation reverts the systemic and molecular changes associated with SOD1(G93A) energy deficit

**Figure 3:**
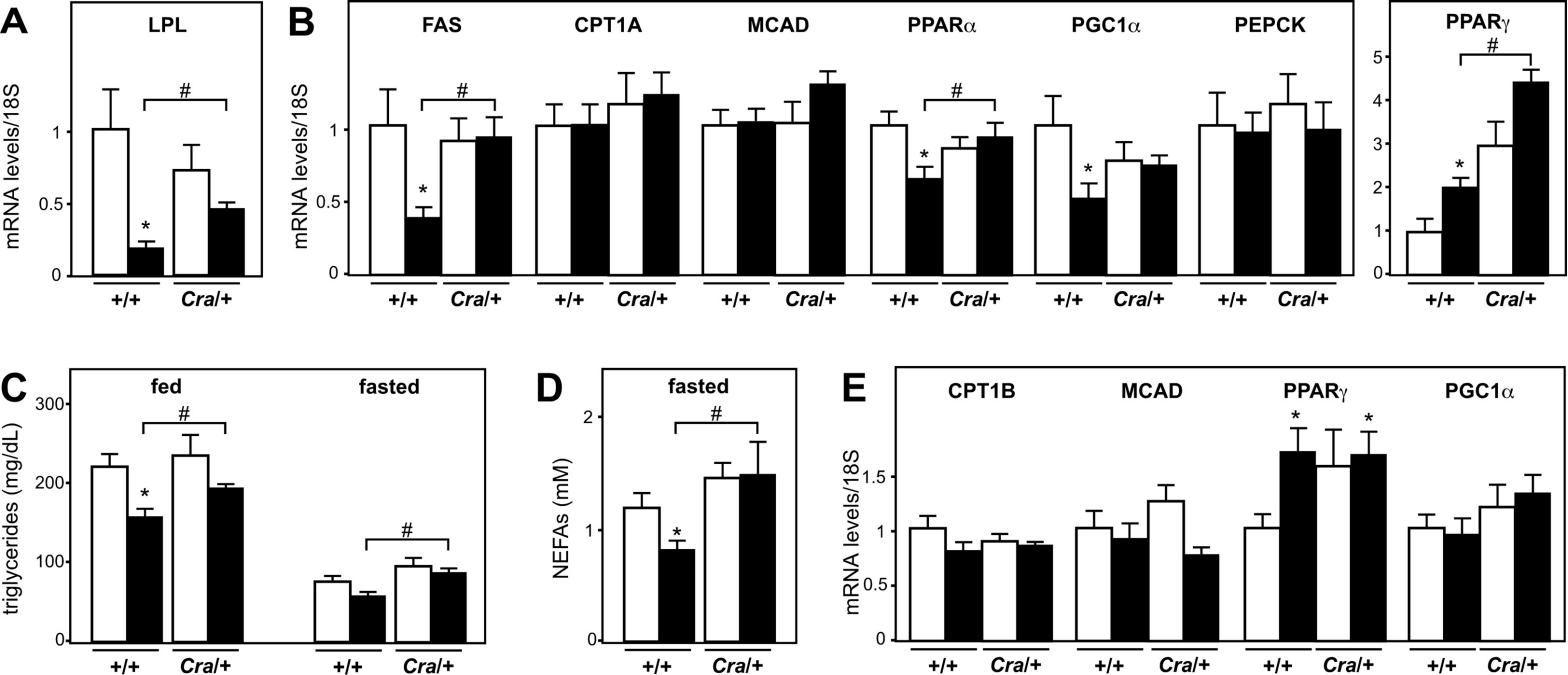
**Dynein mutation reverts the systemic and molecular alterations associated with energy deficit in SOD1(G93A) mice**. A- mRNA levels of lipoprotein lipase (LPL) in the epididimary white fat pad of wild type (+/+) and dynein mutant mice (*Cra*/+) bearing SOD1(G93A) transgene (SOD1m, black columns) or not (Wt, empty columns). *P < 0.05 *versus *Wt; #, p < 0.05 as indicated. Note that the SOD1(G93A)-associated decreased expression of LPL in the EPI is rescued by the dynein mutation in compound SOD1(G93A)/*Cra *mice. N = 9 mice per group. B- mRNA levels of fatty acid synthase (FAS), carnithine palmitoyl transferase 1A (liver form, CPT1A), Medium chain acyl CoA dehydrogenase (MCAD), peroxisome-proliferator activated receptor alpha (PPARα), peroxisome-proliferator activated receptor gamma co-activator 1 alpha (PGC1α), phosphoenolpyruvate carboxykinase (PEPCK) and peroxisome-proliferator activated receptor gamma (PPARγ) in the liver of the same mice than in A. *P < 0.05 *versus *Wt; #, p < 0.05 as indicated. Note that the SOD1(G93A)-associated decreased expression of FAS in the liver is rescued by the dynein mutation in compound SOD1(G93A)/*Cra *mice. N = 9 mice per group. C- Circulating triglycerides levels in the same mice than in A either in fed (right) or fasted conditions (left). Note that the SOD1(G93A) transgene leads to decreased fed triglycerides levels and that this is partially reverted in compound SOD1(G93A)/*Cra *mice. N = 9 mice per group. D- Circulating non-esterified fatty acids (NEFAs) levels in the same mice than in A in fasted conditions. Note that the SOD1(G93A) transgene leads to decreased fasted NEFAs levels and that this is fully reverted in compound SOD1(G93A)/*Cra *mice. N = 9 mice per group. E- mRNA levels of carnithine palmitoyl transferase 1B (muscle form, CPT1A), Medium chain acyl CoA dehydrogenase (MCAD), peroxisome-proliferator activated receptor gamma (PPARγ) and peroxisome-proliferator activated receptor gamma co-activator 1 alpha (PGC1α) in the gastrocnemius muscle of the same mice than in A. *P < 0.05 *versus *Wt. N = 9 mice per group.

The decreased lipid oxidation in mice bearing the *Cramping *mutation might be due to their blunted ability to mobilize adipose stores as previously documented [15]. We thus sought to determine whether the increased fat pad weights also reflected a better metabolic status of the mice. Fatty acid synthase (FAS) expression in the liver and Lipoprotein lipase (LPL) expression in white adipose tissue are well known for their regulated expression as a function of nutritional and hormonal cues [18-21]. Most notably, these two genes are profoundly downregulated by energy deficit, including starvation as well as in SOD1(G93A) mice. Here, the *Cramping *dynein mutation was able to revert these down-regulations in *Cra*/SOD1(G93A) mice (Figure 3A-B). This increased expression of FAS was associated with unchanged expression of beta-oxidation enzymes such as CPT1A and MCAD or gluconeogenesis enzymes such as PEPCK in the liver (Figure 3B). However, expression levels of PPARα and PGC1α, two critical players in the transcriptional control of liver beta-oxidation were decreased by expression of SOD1(G93A) and expression of PPARγ was increased. The *Cramping *dynein mutation reverted PPARα and PGC1α downregulations and potentiated PPARγ upregulation in *Cra*/SOD1(G93A) mice (Figure 3B). A hallmark of energy homeostasis defect in SOD1(G93A) mice is the occurrence of decreased circulating triglycerides after feeding [2]. Consistent with these studies, fed, but not fasted; triglycerides were decreased in SOD1(G93A) mice. The *Cramping *dynein mutation partially compensated for this defect (Figure 3C). Upon fasting, non-esterified fatty acids levels were decreased in SOD1(G93A) mice, as a likely result of increased muscle uptake [2,3], and this was fully reverted by the *Cramping *mutation (Figure 3D). The effect of dynein mutation on metabolic gene expression was not observed in skeletal muscle, in which gene expression of MCAD, CPT1B and PGC1α were unaffected by either SOD1(G93A) or *Cramping *dynein mutation (Figure 3E). Thus, dynein mutation not only compensates for energy deficit, but also reverts systemic and molecular changes associated with SOD1(G93A) energy deficit.

### Dynein mutation increases neuroprotective IGF-1

**Figure 4:**
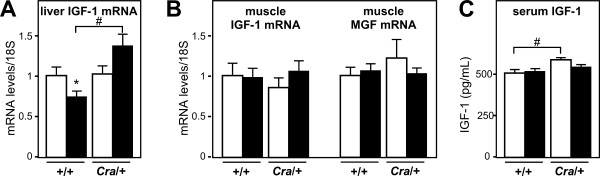
**Dynein mutation increases liver IGF-1 expression**. A- mRNA levels of Insulin-like growth factor (IGF-1) in the liver of wild type (+/+) and dynein mutant mice (*Cra*/+) bearing SOD1(G93A) transgene (SOD1m, black columns) or not (Wt, empty columns) *P < 0.05 *versus *Wt; #, p < 0.05 as indicated. N = 9 mice per group. B- mRNA levels of Insulin-like growth factor (IGF-1) and its muscle specific splice variant mechano-growth factor (MGF) in the gastrocnemius muscle of wild type (+/+) and dynein mutant mice (*Cra*/+) bearing SOD1(G93A) transgene (SOD1m, black columns) or not (Wt, empty columns). N = 9 mice per group. C- Serum IGF-1 levels in wild type (+/+) and dynein mutant mice (*Cra*/+) bearing SOD1(G93A) transgene (SOD1m, black columns) or not (Wt, empty columns) #, p < 0.05 as indicated. Note that circulating IGF-1 levels are increased in dynein mutant mice and that this increase is abolished in compound SOD1(G93A)/*Cra *mice. N = 9 mice per group.

**Figure 5:**
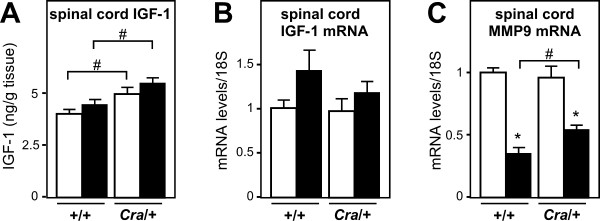
**Dynein mutation increases potentially neuroprotective IGF-1**. A- Spinal cord IGF-1 levels in wild type (+/+) and dynein mutant mice (*Cra*/+) bearing SOD1(G93A) transgene (SOD1m, black columns) or not (Wt, empty columns) #, p < 0.05 as indicated. Note that spinal cord IGF-1 levels are increased in dynein mutant mice and that this increase is stronger in compound SOD1(G93A)/*Cra *mice. N = 9 mice per group. B- mRNA levels of Insulin-like growth factor (IGF-1) in the spinal cord of wild type (+/+) and dynein mutant mice (*Cra*/+) bearing SOD1(G93A) transgene (SOD1m, black columns) or not (Wt, empty columns). N = 9 mice per group. C- mRNA levels of matrix metallo proteinase 9 (MMP9) in the spinal cord of wild type (+/+) and dynein mutant mice (*Cra*/+) bearing SOD1(G93A) transgene (SOD1m, black columns) or not (Wt, empty columns). *P < 0.05 *versus *Wt; #, p < 0.05 as indicated. N = 9 mice per group.

We next turned to define potential underlying mechanisms linking energy deficit and motor neuron survival. One candidate mechanism could involve IGF-1. Circulating IGF-1 is a growth factor mainly produced by the liver, but also, in lesser amounts, in other cell types, including muscle, astrocytes and neurons. IGF-1 has been reported to exert neuroprotective effects [22] and to delay motor neuron loss in SOD1(G93A) mice after administration in an early stage of the disease [23,24]. IGF-1 expression is known to be decreased upon energy deficit [25]. In SOD1(G93A) mice, liver IGF-1 expression was decreased of about 30% (Figure 4A), a situation similar to massive energy deficit triggered by 60% caloric restriction [25]. Consistent with reversal of energy deficit, the *Cramping *dynein mutation completely reverted IGF-1 mRNA downregulation, and even increased it (Figure 4A). Skeletal muscle IGF-1 mRNA levels were unchanged as were mRNA levels of mechano- growth factor, a muscle specific splice variant of IGF-1 [26] (Figure 4B) suggesting that these changes in IGF-1 mRNA levels were restricted to the liver. We next wanted to determine whether hepatic IGF-1 upregulation translated into increased circulating IGF-1. In SOD1(G93A) mice, plasma IGF-1 levels were surprisingly unchanged, suggesting that other mechanisms, including transcription in other cell types or regulated translation of hepatic IGF-1 mRNA occurred [27]. Plasma IGF-1 levels were modestly increased in *Cramping *dynein mutant mice but not in *Cra*/SOD1(G93A) mice (Figure 4C). IGF-1 is poorly but significantly transported throughout the blood brain barrier [28-30] in normal conditions and its entry into the CNS is regulated by neuronal activity [31]. Since the blood brain barrier of SOD1(G93A) mice is disrupted [32,33], it is plausible that an increased fraction of IGF-1 is retained in the spinal cord of *Cra*/SOD1(G93A) mice. Indeed, spinal IGF-1 was increased in *Cramping *dynein mutant mice and this was further enhanced by the SOD1(G93A) transgene (Figure 5A). IGF-1 mRNA levels were unchanged in the spinal cord (Figure 5B) arguing against the proposal that these increased spinal levels of IGF-1 were due to increased local transcription. Last, mRNA levels of MMP-9 were partially restored by the *Cramping *dynein mutation (Figure 5C) consistent with the hypothesis of increased IGF-1 transcytosis upon dynein mutation. Thus, the dynein mutation increases availability of IGF-1 to motor neurons in the spinal cord. The increased systemic production of IGF-1 by the liver might be the source of increased spinal IGF-1. Such a mechanism could account for the neuroprotective effects of the dynein mutation towards SOD1(G93A) pathology.

## Discussion

Our current studies provide evidence that the *Cramping *dynein mutation is able to revert at some point the energy deficit of SOD1(G93A) mice, and to increase IGF-1 levels in the spinal cord of SOD1(G93A) animals. These results, along with previous studies, suggest that two complementary protective pathways are acting in dynein mutant mice to provide the paradoxical protection towards SOD1(G93A) pathology.

### Dynein mutation compensates for energy deficit of SOD1(G93A) mice: a rationale explanation for a seemingly paradoxical observation

This study stems from the seminal observation of a moderate lifespan extension of SOD1(G93A) mice when bearing either *Loa *or *Cra *mutation [6-8]. These studies intiated a flurry of research to explain the seemingly paradoxical observation that two distinct molecular injuries that independently lead to ALS-like disease might yield a better outcome than a single one. Indeed, dynein mutant mice were initially thought to develop motor neuron degeneration [9], and it appeared difficult to understand how mutating dynein, and thus precipitating late onset motor neuron degeneration, might protect against early onset SOD1(G93A)-mediated neurodegeneration. Closer examination of dynein mutant mice however refuted that they displayed motor neuron degeneration [8,10,12]. In the nervous system, these mice display a frank perinatal sensory neuropathy, accompanied by mild striatal atrophy in the absence of neurodegeneration [13]. Furthermore, apart from this neuronal phenotype, dynein mutant mice also develop a number of peripheral defects, especially in brown and white adipose tissues, that are largely reminiscent of striatal degeneration diseases [15]. The peripheral phenotype of dynein mutant mice was strikingly opposite to that of SOD1(G93A) mice. Indeed, while dynein mutant mice accumulate fats [15], SOD1(G93A) mice are leaner and lose white fat pads with disease progression [3]. The origin of this energy deficit is currently unknown but is associated with increased energy expenditure. Moreover, compensating energy deficit through high fat feeding alleviates motor neuron degeneration [3]. Importantly, these observations are of great relevance for the human pathology since lipemia is positively correlated with survival of ALS patients and energy status appears as a widely documented prognostic factor [1]. In our hands, crossbreeding of dynein mutant mice with SOD1(G93A) mice yielded very similar effects as high fat feeding, by increasing energy storage. Increased RQ during nocturnal period suggests indirectly increased beta-oxidation, while our gene expression analysis are consistent with increased diurnal lipogenesis in compound *Cra */SOD1(G93A) mice. Such a metabolic picture is fully consistent with the observed improved energy status. Thus, a straightforward interpretation would be that the dynein mutant mediated injury compensates mutant SOD1 injury through its effect on energy homeostasis, thereby alleviating neurodegeneration.

### Potential involvement of IGF-1 in dynein mutant mediated protection

We further provide mechanistic insights into how the dynein mutant peripheral phenotype might provide protection to motor neurons. Liver IGF-1 expression is under the control of nutritional cues, and is for instance decreased in starved animals [25]. Consistent with their energetic status, we observed downregulation of IGF-1 in SOD1(G93A) liver, and this was fully reverted by dynein mutation. It should be noted however that SOD1(G93A) showed only limited analogy with starved animals since liver expression of genes such as PPARα or PGC1α showed regulations opposite to those observed in starved animals. In double mutant mice, while there were no changes in circulating IGF-1, we observed increased spinal IGF-1. A similar trend was observed between wild type and SOD1(G93A) animals. One may consider that the increased hepatic production of IGF-1, due to the reversal of the energy deficit of SOD1(G93A) mice, is directed towards the nervous system, and thus motor neurons, of SOD1(G93A) mice. This is consistent with our results at the different levels at which we investigated IGF-1 (liver, skeletal muscle, plasma and spinal cord). This interpretation is further in line with modified expression of IGF-1 receptors and IGF-1 binding proteins that occur in SOD1(G93A) mice motor neurons [34,35]. It should be noted here that circulating, liver derived IGF-1 is able to cross the blood brain barrier (BBB) and counteract age-related decline in cognitive functions [36,37]. This natural entry might even be potentiated by leakage of the BBB during ALS disease [32,33]. Also, IGF-1 transcytosis has been shown to be dependent upon neuronal activity, which leads to increased MMP-9 activation and cleavage of IGF binding proteins [31]. Interestingly, we previously observed increased activity of MMP-9 in SOD1(G93A) spinal cord [38] and others have documented decreased levels of IGF binding proteins [35] in SOD1(G93A) CNS. Decreased MMP9 mRNA levels in sick SOD1(G93A) mice is not at odds with previous results since we and others have shown that MMP9 activity actually peaks before onset and then decreases [38,39]. In all, our results support that IGF-1 transcytosis might be increased in SOD1(G93A), and to an even greater extent in *Cra*/SOD1(G93A) mice.

Our study does not directly address whether increased IGF-1 is responsible for the extension in lifespan. This is however plausible as most studies observed protective effect of increasing IGF-1 in SOD1(G93A) mice [23,24]. A direct positive impact on neuromuscular junctions from circulating IGF-1 cannot be excluded since muscle-restricted expression of IGF-1 is able to stabilize neuromuscular junctions and delay motor neurons death of SOD1(G93A) G93A mice [24]. Also, IGF-1 is able to provide protection to mitochondria even at low doses in aging rats [40,41] through a boost in mitochondrial biogenesis and our results are thus consistent with the recent observation of mitochondrial protection in SOD1(G93A) mice by dynein mutation [16]. In human clinical trials however, IGF-1 did not achieve efficacy in ALS clinical trials [42]. This lack of efficacy might be due to inappropriate targeting of IGF-1 to motor neurons (see below). Alternatively, IGF-1 might be acting in combination with other still unknown circulating factors to provide full protection in dynein mutant mice. In all, we propose that circulating factors modified by the reversal of energy deficit, and between these, IGF-1, contribute to the protection offered by dynein mutation.

### Mutant dynein mediated protection may rest on two complementary biological events

Several research groups, including ours, have previously shown that dynein *Cra *and *Loa *mutations were able to increase the lifespan of SOD1G93A mice. Several mechanisms have been proposed to explain the underlying mechanisms of mutant dynein protection towards mutant SOD1 pathogenesis. To conclude, we would like here to discuss critically these different hypotheses in the light of our results and provide a working model summarizing these different potential mechanisms.

In the first study, Kieran et al. [6] suggested a cell autonomous effect of dynein mutation in motor neurons and proposed that the defect in retrograde transport triggered by mutant dynein counterbalanced the one due to SOD1(G93A) in the anterograde direction, and thereby restoring axonal homeostasis. This cell autonomous protective effect of dynein mutation is in line with results obtained by Teuling and collaborators [43] that used neuron-specific overexpression of an N-terminal deleted form of BicD2 to modulate dynein/cargo interaction [44]. Interestingly, BicD2 overexpression in neurons disrupted retrograde axonal transport, delayed the SOD1 aggregates retrograde transport toward the cell center and increased survival of SOD1 G93A mice [43]. However, BicD2 overexpression might also have a number of dynein independent effects, including on kinesin-mediated transport [45] Pan-neuronal overexpression of BicD2 might also lead to impairment of neuronal physiology in neurons other than motor neurons, including in proprioceptive neurons (see below). Thus, the hypothesis of the restoration of axonal homeostasis by double blockade of both anterograde and retrograde directions of axonal transport remains unproven.

**Figure 6:**
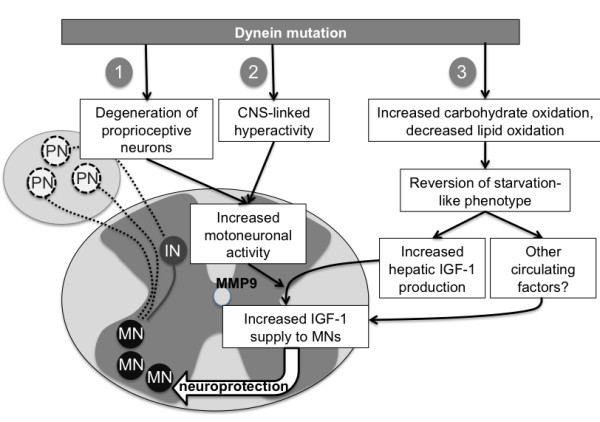
**a working model for dynein mutant protection against SOD1-ALS**. We propose that dynein mutations provide neuroprotection by converging paths. First, degeneration of proprioceptive neurons (PN) modify network activity (1) by decreasing direct glutamatergic input to motor neurons (MN), but also to inhibitory interneurons (IN). Second, CNS-linked hyperactivity (2) might on its own lead to increased motoneuronal activity, and MMP9 activation. Last, by modifying energy metabolism and favoring carbohydrate oxidation over lipid oxidation (3), dynein mutation reverts the energy deficit of SOD1(G93A) mice, thereby increasing IGF-1 production in the liver. This increased IGF-1 production is able to enter the CNS due to MMP9 activation. Other circulating factors might also increase IGF-1 supply to motor neurons.

The complex phenotype of dynein mutant mice render very plausible that dynein mutation could also act at multiple levels besides motor neurons. First, dynein mutation is likely to reduce glutamate excitotoxicity through the degeneration of proprioceptive glutamate inputs to motor neurons [8]. Although this hypothesis is initially attractive, type Ia afferents that degenerate in dynein mutant mice stimulate also inhibitory interneurons to perform reciprocal inhibition [46]. The exact effect of dynein mutation on excitotoxic load on motor neurons thus remains to be investigated. Besides proprioceptive afferents, motoneuronal activity is likely to be increased in dynein mutant mice due to increased spontaneous activity [13]. Our current findings lead us to propose that the protective dynein mutations, such as *Legs at odd angles *and *Cramping*, also impact bioenergetics' mechanisms and revert the energy deficit of SOD1(G93A) mice. In the compound mice, this leads to increased production of IGF-1. We propose that the proprioceptive degeneration and CNS linked hyperactivity converge to modify neuronal activity and facilitate the net transcytosis of IGF-1 in the vicinity of motor neurons, thus achieving neuroprotection. This working model is summarized in Figure 6.

## Conclusions

These findings suggest that the protection against SOD1(G93A) offered by the *Cramping *mutation in the dynein gene is, at least partially, mediated by a reversal in energy deficit and increased IGF-1 availability to motor neurons. Our study provides a rationale explanation to increased survival in double dynein mutant/SOD1(G93A) mice and suggests that the neuroprotection results from dynein mutant phenotype in various tissues.

## Methods

### Animals

Heterozygous *Cra*/+ females were crossed with SOD93A males and were identified by tail DNA genotyping for the human transgene SOD1 (G93A) and the *Cra *mutation as described previously [7,9]. Experiments were performed with littermates mice with 9 mice per group. Mice were maintained at 23°C with a 12h light/dark cycle and had food and water *ad libitum*. All animal experiments were performed under the supervision of authorized investigators and followed current EU regulations. Animals were treated in accordance with the European Union guide for the care and the use of animals in research (commission June 18th 2007, 2007/526/CE). LD has been allowed to perform mouse experiments (agreement A67-266, direction des services vétérinaires, Strasbourg, Bas-Rhin, France).

### Indirect calorimetry

We measured O_2 _consumption and CO_2 _production by using an open-circuit indirect calorimetry system (Sable systems, Las Vegas, USA). Concentrations of O_2 _and CO_2 _in the outgoing air were successively measured in five different cages. The system was rinsed for 90 s between each measurement. Final values of gas concentrations were the mean of 10 measures obtained during 40 s. Each cage was sampled every 11 min, and one cage was left vacant as reference of ambient gas concentrations. Measurements were performed continuously over 23 1/2 h, a 30-min period being required for calibration of the O_2 _and CO_2 _analyzers. In total, 127 measures were collected per day and mouse. The average of the five lowest values of O_2 _consumption was considered as resting energy expenditure. Energy expenditure was obtained by using an energy equivalent of 20.1 J/ml O_2_. The respiratory quotient was the ratio of CO_2 _production over O_2 _consumption.

### RT-qPCR

RNA were extracted by using Trizol^® ^(Invitrogen, Cergy-Pontoise, France). Real time RT quantitative PCR was performed with one microgramme of total RNA as described [13]. PCR analysis was carried out on a Bio-Rad iCycler System using iQSYBR Green Supermix. A specific standard curve was performed in parallel for each gene, and each sample was quantified in duplicate. PCR conditions were 3 min at 94°C, followed by 40 cycles of 45 s at 94°C and 10 s at 60°C. The relative levels of each RNA were normalized to 18S RNA levels. Oligonucleotide sequences are reported in the table 1.

**Table 1:** Oligonucleotides sequences

Name of the gene	Forward primer	Reverse primer
18S	TCTGATAAATGCACGCATCC	GCCATGCATGTCTAAGTACGC
AchRalpha	CCACAGACTCAGGGGAGAAG	AACGGTGGTGTGTGTTGATG
CPT1a	GCTGTCAAAGATACCGTGAGC	TCTCCCTCCTTCATCAGTGG
CPT1b	TGCCTTTACATCGTCTCCAA	GGCTCCAGGGTTCAGAAAGT
FAS	TCTGCAGAGAAGCGAGCATA	CCCAGAGGGTGGTTGTTAGA
IGF-1	GCTTGCTCACCTTTACCAGC	AAATGTACTTCCTTCTGGGTCT
MCAD	TGTCGAACACAACACTCGAAA	CTGCTGTTCCGTCAACTCAA

### Biochemical assays

Mouse IGF-1 levels were measured using Mouse/Rat IGF-I Quantikine ELISA Kit (R&D systems), using manufacturer's instructions. Plasma triglycerides and NEFAs were measured using Randox kits using manufacturer's instructions.

### Statistical Analysis

Statistical comparisons were accomplished with the unpaired Student t test, unless otherwise indicated, or ANOVA followed by the post hoc Newman-Keuls multiple comparisons test using PRISM version 2.0a software (GraphPad, San Diego).

## List of abbreviations

AchRα: alpha subunit of the nicotinic cholinergic receptor; ALS: amyotrophic lateral sclerosis; BBB: blood brain barrier; BicD2: bicaudal D2; CNS: central nervous system; *Cra*: *Cramping *mutation of the Dyn1hc1 gene; FAS: fatty acid synthase; IGF-1: Insulin-like growth factor 1; LPL: lipoprotein lipase; SOD1: copper-zinc superoxide dismutase.

## Competing interests

The authors declare that they have no competing interests.

## Authors' contributions

AF performed animal handling and follow up, participated in the indirect calorimetry experiments, performed qPCR assays and most of the biochemical assays; JE performed qPCR assays and some biochemical assays, analyzed indirect calorimetry results and drafted the manuscript; HO performed indirect calorimetry experiments; YL performed animal follow up and analyzed results; BS performed animal handing, genotyping and analyzed results; ACL and JPL conceived of the study, participated in its design and helped to draft the manuscript; LD conceived of the study, designed and coordinated the study and drafted the manuscript. All authors read and approved the final manuscript.
